# Reply to Emv2, the only endogenous ecotropic murine leukemia virus of C57BL/6J mice

**DOI:** 10.1186/1742-4690-9-24

**Published:** 2012-03-22

**Authors:** Kang-Hoon Lee, Kiho Cho

**Affiliations:** 1 Departments of Surgery, University of California, Davis and Shriners Hospitals for Children Northern California, Sacramento, CA 95817, USA

## Abstract

This correspondence was written in response to the comments by Young *et al. *Following careful evaluation of the relevant dataset, each of the points brought up by Young *et al. *has been addressed in this response. We anticipate this will clarify our findings regarding ERV_mch8_, an ecotropic endogenous retrovirus that was shown to have cerebellum-specific and age-dependent expression patterns in C57BL/6J mice.

## Correspondence

The authors of the paper by Lee *et al *[1]. appreciate the interest shown and comments by Young *et al *[2].

Young *et al. *contend that Emv2 is the only endogenous ecotropic murine leukemia virus in C57BL/6J mice, although not annotated on the current NCBI reference genome assembly, Build 37.2, and maps to the distal region of chromosome 8 based on previous genetic studies.

**Figure 1:**
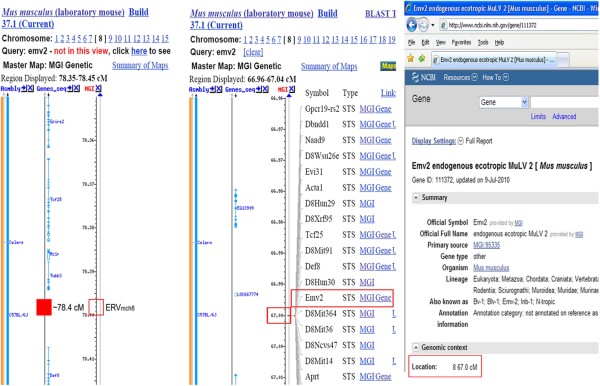
**Screenshots (as of November, 2010) of the chromosomal regions of ERV_mch8 _and Emv2 loci in the NCBI mouse genome database, Build 37.1**. Both NCBI and MGI annotated the Emv2 locus on the C57BL/6J mouse chromosome 8 at the ~67 cM region where no sequence similar to mouse endogenous retroviruses was present. However, within the chromosome 8 region of 125958431-125949704 (~78.4 cM), the full/complete ERV_mch8 _sequence was retrieved.

As stated in the paper by Lee *et al.*, the Emv2 locus was annotated in the NCBI database Build 37.1 when analyzed around November 2010. At that time, the annotated Emv2 locus did not contain any sequences similar to mouse endogenous retroviruses. Interestingly, a survey of the latest NCBI database (Build 37.2) for the Emv2 locus on December 08, 2011, in response to the correspondence by Young *et al.*, yielded no annotated information about the Emv2 locus, and it appears that the previous annotations may have been removed. During the course of our analysis regarding the Emv2 locus in the C57BL/6J mouse genome, screenshots of the data were captured and recorded, and revealed the annotation of the Emv2 locus on chromosome 8, which is distantly located from the ERV_mch8 _locus (Figure 1). Although the paper by Lee *et al. *uses Build 37.1 to show the annotation for Emv2, this annotation for Emv2 appears as far back as 2006 (NCBI, when reverting to the previous build version 36.1).

This was cited in the paper by Lee *et al.*:

*In addition, Emv2 is located/annotated at 67.0 cM, ~11.4 cM upstream of the ERV_mch8 _locus (~78.4 cM), according to a survey of the NCBI map viewer (*http://www.ncbi.nlm.nih.gov/projects/mapview)... *Unexpectedly, we were unable to retrieve the nucleotide sequence, which is presumed to be the Emv2 provirus, from the Emv2 locus annotated in the ... However, it is still a possibility that ERV_mch8 _shares the same locus on chromosome 8-qE1 region with Emv2 with an assumption that the NCBI annotation information regarding the Emv2 locus needs to be revised*.

As indicated in the paper by Lee *et al.*, we were not able to find the "full/complete" sequence information from the NCBI database using the keyword "Emv2", and it was unclear why there was no "full/complete" sequence information deposited into the database when substantial work in regard to Emv2 has been reported. When preparing the manuscript, a thorough survey of relevant scientific publications for Emv2 also yielded neither "full/complete" nor partial sequence information.

We suggest a further study is needed to confirm whether the ERV_mch8 _locus indeed matches the Emv2 locus. Importantly, the difference in distance between the annotated Emv2 locus (chromosome 8 at ~67 cM region) and the ERV_mch8 _locus (chromosome 8 at ~78.4 cM region) needs to be clarified (Figure 1). In addition, it appears that the result from Young *et al.*'s "*in silico *Southern blot" analysis referencing the ERV_mch8 _sequence may not be sufficient to confirm that ERV_mch8 _is Emv2. A well-focused experiment is needed to determine whether the previously reported Emv2 locus matches to the ERV_mch8 _locus. For example, to help determine whether ERV_mch8 _is indeed Emv2, a set of Southern blot analyses can be performed using the genomic DNAs from various mouse strains, including the mouse strains reported in the studies referenced by Young *et al.*, with probes designed from the "full/complete" ERV_mch8 _sequence, in addition to the pEco probe. It will be very helpful if the original DNAs, which were used for the Emv studies referenced by Young *et al.*, are still available. In addition, it may be necessary to clone and sequence the ERV_mch8_/Emv2 locus in these mouse strains to examine whether there are any sequence polymorphisms among the presumed loci in different mouse genomes.

Young *et al. *contend that the terminology "novel ecotropic provirus" and labeling the virus as having an "intact coding potential" is misleading. Throughout the manuscript, the word "novel" was used only once for the endogenous retrovirus (ERV_mch8_) in the title of Figure 1, primarily because, as previously stated, the NCBI Emv2 annotation locus was completely different from the ERV_mch8 _locus (Please refer to Figure 1 of this response.) Furthermore, the title of the manuscript does not contain the word "novel." In fact, the paper suggests that the NCBI annotation of Emv2 may need revision. Additionally, the phrase "intact coding potential" was used to indicate an "intact open reading frame" is present, without suggesting a precise function of the protein to be coded. The abstract of the paper by Lee *et al. *explains:

*It appears that ERV_mch8 _shares the same genomic locus with a replication-incompetent MuLV-ERV, called Emv2; however, it was not confirmed due to a lack of relevant annotation and Emv2 sequence information*.

Young *et al. *also advise that different reference virus choices should be made for phylogenetic classification. We appreciate Young *et al.*'s suggestion in regard to the choice of reference sequences; however, our analyses focused on the evaluation of relatedness at the nucleotide level, not at the virus level.

Additionally, Young *et al. *remind us that previous studies by Jenkins *et al.*, using a Southern hybridization method, found Emv2 only on C57BL/6J and a few related mouse strains. Young *et al. *also suggest that the PCR approach employed by Lee *et al. *uses "*primers that are wrongly presumed specific for the 'ERV_mch8_' sequence, [and] found an ecotropic provirus on chromosome 8 in a wide variety of strains."*

The primer set used in the paper by Lee *et al. *was designed to amplify a unique region from the end of the *env *gene to the middle of the LTR of ERV_mch8 _in the C57BL/6J mouse genome. *In silico *PCR (UCSC) retrieved only one locus for ERV_mch8_, which is located on chromosome 8 of the C57BL/6J mouse genome and the only copy of an ecotropic MuLV found in that genome. The data presented in the paper by Lee *et al. *intend to only show the general distribution of the 'ERV_mch8 _sequence' throughout the genomes of 57 mouse strains and do not specifically refer to an ERV_mch8 _sequence on chromosome 8 (since the copy number or locations can vary depending on the strain). As stated in the results section of the paper by Lee *et al.*, the variability of size and intensity of these bands suggests "*polymorphisms in the sequences and/or copy numbers*."

It should also be noted that both the Southern blot and PCR protocols share a specific step which is "nucleic acid" to "nucleic acid" hybridization. As such, both protocols harbor the potential for false identification of the intended target DNAs. Our laboratory also routinely includes relevant RT-negative controls for RT-PCR analyses.

We hope that the responses by Lee *et al. *clarify the comments brought up by Young *et al. *We anticipate this correspondence will clarify our findings regarding ERV_mch8_, an ecotropic endogenous retrovirus that was shown to have cerebellum-specific and age-dependent expression patterns in C57BL/6J mice.

## Competing interests

The authors declare that they have no competing interests.

## Authors' contributions

The authors contributed equally to this article.
